# Methylprednisolone alone or combined with cyclosporine or mycophenolate mofetil for the treatment of immune‐mediated hemolytic anemia in dogs, a prospective study

**DOI:** 10.1111/jvim.17122

**Published:** 2024-07-03

**Authors:** Chiara Agnoli, Michele Tumbarello, Kateryna Vasylyeva, Carola S. Selva Coddè, Erika Monari, Marta Gruarin, Roberta Troìa, Francesco Dondi

**Affiliations:** ^1^ Department of Veterinary Medical Sciences Alma Mater Studiorum‐University of Bologna Bologna Italy; ^2^ VPG, Unit 8 Temple Point Leeds UK

**Keywords:** dog, glucocorticoids, hematological response, IMHA, immunosuppression, therapy

## Abstract

**Background:**

Benefit of adding a second‐line immunosuppressive drug to glucocorticoids for the treatment of non‐associative immune‐mediated hemolytic anemia (naIMHA) in dogs has not been defined prospectively.

**Hypothesis/Objectives:**

Evaluate the effectiveness of different immunosuppressive protocols in naIMHA dogs.

**Animals:**

Forty‐three client‐owned dogs.

**Methods:**

Open label, randomized, clinical trial. Dogs were treated with methylprednisolone (M‐group), methylprednisolone plus cyclosporine (MC‐group) or methylprednisolone plus mycophenolate mofetil (MM‐group). Dogs were defined as responders by disappearance of signs of immune‐mediated destruction and hematocrit stabilization. Frequency of responders was compared between M‐group and combined protocols (MC and MM‐group evaluated together), and among the 3 different therapeutic groups at 14 (T14), 30 (T30), 60 (T60) days after admission. Frequency of complications, length of hospitalization and relapse were also compared. Death rate was evaluated at discharge, T60 and 365 (T365) days.

**Results:**

Proportion of responders was not significantly different between M‐group and combined protocols (MC and MM‐groups), nor among the 3 therapeutic groups at T14, T30, and T60 (*P > .17*). Frequency of relapse, complications, and length of hospitalization were not significantly different between M‐group and dogs treated with combined protocols, nor among the 3 treatment groups *(P > .22).* Death was significantly more common only for MM‐group compared with MC‐group at T60 (+42.8%; 95% CI: 11.5–67.4; *P* = .009), and at T365 (+50%; 95% CI: 17.5–73.2; *P* = .003).

**Conclusions and Clinical Importance:**

Combined immunosuppressive therapy did not improve hematological response in naIMHA.

**Abbreviations**
ACVIM: American College of Veterinary Internal Medicine
aIMHA: associative immune‐mediated hemolytic anemia
APPLE_fast_
: acute patient physiologic and laboratory evaluation fast score
aPTT: activated partial thromboplastin time
CHR: complete hematological recovery
CRP: C‐reactive protein
HCT: hematocrit value
IMHA: immune‐mediated hemolytic anemia
MAT: microscopic agglutination test
MMF: mycophenolate mofetil
naIMHA: non‐associative immune‐mediated hemolytic anemia
NR: non‐responders
PP: per protocol
PHR: partial hematological recovery
PRBCs: packed red blood cells
RBCs: red blood cells
SAT: saline agglutination test
UPC: urine protein‐to‐urine creatinine ratio

## 1 INTRODUCTION

Immune‐mediated hemolytic anemia (IMHA) is the most common autoimmune disease in dogs^1^ and is based on a type‐II hypersensitivity reaction in which opsonized red blood cells (RBCs) are destroyed by phagocytosis, activation of the complement cascade, or both.^1^ Recently, IMHA has been classified into associative (aIMHA) and non‐associative IMHA (naIMHA) according to the presence or absence of a trigger for RBCs immune‐mediated destruction, respectively.^2^ Despite many aspects of the disease are well characterized, IMHA still represents a medical challenge, given the severity of clinical signs, the common need for intensive care support and the high case fatality rate, up to 50%.^3^, ^4^, ^5^, ^6^

The treatment of IMHA involves halting the ongoing RBCs destruction caused by the immune system and includes different immunosuppressive approaches combined with supportive therapies. Glucocorticoids remain the cornerstone of the treatment because of their effects on both the humoral and cell‐mediated immune response. Many dogs can be managed satisfactorily with steroids alone, while others develop life‐threatening IMHA forms or severe steroid‐induced adverse events, thereby potentially requiring the introduction of additional immunosuppressive drugs.^4^ Among second‐line drugs, previous studies have suggested the use of mycophenolate mofetil (MMF), suppressor of de novo purine biosynthesis in lymphocytes, or cyclosporine, a calcineurin inhibitor.^7^ A clear benefit of adding these drugs to a standard steroid protocol has not been established yet,^8^, ^9^, ^10^, ^11^ and to the best of our knowledge, prospective randomized studies comparing different treatments are lacking.

The primary aim of this study was to document the effectiveness of 3 different immunosuppressive protocols on the achievement of hematological recovery in dogs with spontaneously occurring naIMHA. Secondary aims were to assess length of hospitalization, frequency of complications and relapse, and case fatality rate in the treatment groups. We hypothesized that dogs undergoing to a combined immunosuppressive treatment would experience a shorter time to hematological remission and hospital discharge, lower rate of relapse and death than dogs treated with glucocorticoid monotherapy.

## 2 MATERIALS AND METHODS

### 2.1 Study design, case selection

This study was designed as an open label, unblinded, randomized controlled clinical trial. Dogs with newly diagnosed naIMHA admitted to the Veterinary University Hospital of the University of Bologna, between April 2018 and October 2020, were consecutively enrolled. Included dogs had to undergo diagnostic investigations to establish a diagnosis of naIMHA including complete history, physical examination, hematology, serum biochemistry, urinalysis, vector‐borne disease testing, antinuclear antibody testing, thoracic radiography, abdominal ultrasonography, and fine‐needle aspiration of liver and spleen. The study was approved by the local Scientific Ethical Committee for Animal Testing, and written consent was obtained from owners. Diagnosis of naIMHA was based on (a) presence of anemia defined as a hematocrit value (HCT) <37%; (b) at least 1 of the following^12^, ^13^: (1) positive direct Coombs' test^14^; (2) positive saline agglutination test (SAT)^15^; (3) presence of spherocytes on the blood smear (≥5 spherocytes/×1000)^16^; (c) 1 or more signs of hemolysis (hyperbilirubinemia, bilirubinuria, without hepatic or posthepatic diseases, or both; hemoglobinemia; hemoglobinuria); (d) no evidence of underlying disease or administration of drugs recognized as potential triggers for associative IMHA, as previously reported.^2^ Transfusions or immunosuppressive treatments administered before the admission represented an exclusion criteria.^4^, ^17^, ^18^

Data evaluated upon admission (T0) included signalment, history, clinical findings, clinicopathological data (CBC, SAT, direct Coombs' test, biochemistry, coagulation analyses, urinalysis, antinuclear antibody test, and diagnostic tests for *Leishmania infantum*, *Ehrlichia canis*, *Anaplasma phagocytophilum*, *Borrelia burgdorferi, Leptospira* spp., *Babesia* spp., and *Dirofilaria immitis*), and imaging findings (thoracic radiographs and abdominal ultrasound). Fine‐needle aspiration of liver and spleen, regardless of their sonographic appearance, were also performed within 48 h from admission in all cases to rule out underlying diseases associated with IMHA. Disease severity was assessed using the acute patient physiologic and laboratory evaluation fast score (APPLE_fast_) and the canine hemolytic anemic objective score (CHAOS), as previously reported.^19^, ^20^ Diagnostics were repeated during the first year of treatment after a schedule determined a priori (Table S1). Further investigations (as adjunctive clinicopathological or imaging evaluation) were made whenever deemed necessary.

### 2.2 Study groups, treatment, and complications

Upon admission, dogs received an initial intravenous dose of methylprednisolone sodium succinate (Solu‐Medrol Vet, Zoetis Italia S.r.l., Rome, Italy) at 2 mg/kg q12h IV for the first 48 h, afterwards each dog was randomly assigned to 1 of 3 different therapeutic protocols using an online available number service website.^21^

Treatment protocols were as follows: (1) methylprednisolone (Solu‐Medrol Vet or Medrol Vet, Zoetis Italia S.r.l., Rome, Italy) administered at a target dose of 1 mg/kg q12h IV or PO (M‐group); (2) methylprednisolone administered at a target dose of 1 mg/kg q12h IV or PO, and oral cyclosporine (Atoplus, Novartis animal Health S.p.a, Origgio, Italy) given at a target dose of 2.5 mg/kg q12h (MC‐group); (3) methylprednisolone administered at a target dose of 1 mg/kg q12h IV or PO and oral mycophenolate mofetil (Cellcept, Roche Registration Limited, Welwyn Graden City, Hertfordshire, UK) given at a target dose of 7.5 mg/kg q12h PO and compounded for individual dogs (MM‐group). Regardless of the assigned group, on day 24 the target dose of methylprednisolone was tapered gradually as follows: 0.75 mg/kg q12h until day 45; 0.50 mg/kg q12h from day 46 to day 66; 0.25 mg/kg q12h from day 67 to day 87, q24h from day 88 to day 101 and finally every other day from day 102 to day 120, when methylprednisolone treatment was withdrawn. A faster reduction in the dose was carried out in dogs experiencing severe related adverse effects (eg, severe polyuria and polydipsia, calcinosis cutis).

After 5 months of treatment, for dogs in the MC‐group cyclosporine was reduced to a target maintenance dose of 2.5 mg/kg q24h, whereas for dogs in the MM‐group mycophenolate mofetil was reduced to a target maintenance dose of 7.5 mg/kg q24h. Both drugs were stopped after 6 months of treatment.

Dogs included in the M‐group that after 2 weeks of treatment showed no hematological improvement (HCT value was equal to or lower than the admission HCT value and positive Coombs' test, or persistent positive SAT, or presence of spherocytes) and were still dependent on packed red blood cells (PRBCs) transfusions were removed from the study and allowed to receive other drugs. These dogs were included in the admission demographics and in the analysis of clinical and clinicopathological data but were excluded from the endpoints assessment for treatment protocol comparison, excepting the intention‐to‐treat analysis of primary outcome, as described below.

Supportive care was standardized whenever possible and recorded for all animals. Pending the results of the tests for infectious diseases, empirical antimicrobial therapy with doxycycline (Vibravet, Zoetis Italia S.r.l., Roma, Italy, at 10 mg/kg q24h) was administered to dogs considered to have a high risk of vector‐borne exposure; furthermore, dogs with direct or indirect ultrasound signs of gastrointestinal ulceration,^22^, ^23^ as well as those with evidence of gastrointestinal bleeding (eg, melena), received omeprazole at 0.7 mg/kg q24h (Omeprazolo, Mylan generics Italia, Milan, Italy).^24^ Dogs with platelet count >30 000/μL were treated with subcutaneous unfractionated heparin (Calciparina, Italfarmaco S.p.a, Milan, Italy) at 150 UI/kg q6h and oral clopidogrel (Plavix, Sanofi Aventis group, Paris, France), at 2 mg/kg q24h. Unfractionated heparin dose was adjusted based on activated partial thromboplastin time (aPTT) monitoring in accordance with recent guidelines.^4^, ^25^, ^26^ Heparin and clopidogrel were discontinued after a period of 15 and 120 days, respectively.

Fresh frozen plasma and PRBCs transfusions were administered based on case‐by‐case anemia tolerance, or the presence of active bleeding. Once discharged, dogs were treated with supportive medication, based on individual needs.

Complications occurring during the treatment period were classified as IMHA‐related (anemia‐induced tissue hypoxia and thromboembolic events) or treatment‐related (infections, or signs of iatrogenic hyperadrenocorticism). Specifically, hypoxic events were defined as a symptomatic reduction of oxygen supply to tissues directly attributable to the severity of anemia (eg, weakness, tachypnea, lethargy, syncope).^27^ Thromboembolic events included venous and arterial thrombosis and pulmonary thromboembolism. These events were suspected in case of consistent clinical, laboratory and imaging findings, such as acutely increased respiratory rate and effort, hypoxemia, increased alveolar‐to‐arterial oxygen gradient, supportive radiographic findings, or direct evidence of arterial or venous thrombosis by means of clinical examination and ultrasound.^28^, ^29^ Infectious complications were defined as the presence of a septic focus documented by cytology, microbiology, or both, as reported.^30^, ^31^ Clinical (eg, polyuria and polydipsia, polyphagia, excessive panting, muscle weakness or atrophy, haircoat changes, and skin abnormalities) and clinicopathological (eg, increased ALP, and GGT activity, hyperlipidemia and hyperglycemia) changes caused by the sustained use of glucocorticoids were defined as iatrogenic hyperadrenocorticism.^32^, ^33^

Complications were classified as minor (mild or subclinical signs, adversely affecting the dog's quality of life, on the owner's compliance, or both), and major (life‐threatening conditions).

### 2.3 Hematological recovery, relapse, and death

Hematological recovery was assessed at day 14 (T14), 30 (T30), and 60 (T60), after inclusion in the study. Complete hematological recovery (CHR) was defined by HCT >37%, negative SAT, absence of spherocytosis, normal total bilirubin concentration and absence of bilirubinuria (defined as >1+ urine dipstick test for bilirubin).^34^ Partial hematological recovery (PHR) was defined as persistency of anemia, however with a HCT increase compared with admission, negative SAT, and absence of spherocytosis, hyperbilirubinemia, and bilirubinuria. Dogs achieving CHR or PHR were classified as responders, while all other dogs as non‐responders (NR).

Relapse was defined as HCT decrease of at least 10%, in addition to ≥1 signs of immune‐mediated RBCs destruction, with a concurrent increase in total bilirubin concentration and presence of hemoglobinuria, bilirubinuria, or both.^35^ Case fatality rate was measured at discharge, at day 60 (T60) and at day 365 (T365), after the enrollment.

### 2.4 Clinicopathological evaluation

Blood samples were collected by venipuncture using a vacuum collection system (Vacutest Kima Srl, Arzergrande, Italy). All blood and urine samples were analyzed within 2 h after collection.

A CBC was carried out by using an automated hematology system (ADVIA 2120, Siemens Healthcare Diagnostics, Tarrytown, NY). Blood smear microscopic evaluation was performed after May‐Grünwald‐Giemsa staining (Merk KGaA, Darmstadt, Germany). Presence and enumeration of spherocytes, and abnormal RBCs morphology were recorded. SAT was performed by washing RBCs 3 times with saline, as previously reported.^15^ The direct antiglobulin test (Lab test Alvedia, Limonest, France) was performed within 4 h after blood collection, following the manufacturer's instructions.

Severity of anemia was graded as follows: mild (HCT 30%–37%), moderate (HCT 20%–29%), severe (HCT 13%–19%), and very severe (HCT <13%).^36^ Anemia was defined as regenerative if the reticulocyte number was >120 000/mm^3^.^37^ Presence of hemolysis was detected by visual examination of the plasma.^38^

Biochemistry was performed by using an automated analyzer (OLYMPUS AU480, Olympus/Beckman Coulter, Brea, California). Blood lactate concentration (Lactate Scout+, EKF Diagnostic, Barleben, Germany), prothrombin time, aPTT, fibrinogen (Thromborel S, Dade Actin, Multifibren U, BFT II Analyzer, Siemens Healthcare GmbH, Erlangen, Germany), urinalysis including urine specific gravity measurement by refractometer, (American Optical, Buffalo, New York), dipstick examination (Combur Test, Urisys 1100 Urine Analyzer, Roche Diagnostics, Mannheim, Germany) and microscopic evaluation of urine sediment, were assessed. Urine protein‐to‐creatinine ratio quantification was performed (Urinary/CSF Protein OSR6170; Creatinine OSR6178, Olympus/Beckman Coulter, O'Callaghan's Mills, Ireland), in not pigmented urine specimens.

Immunofluorescence antibody tests for *L. infantum*, *E. canis*, and *A. phagocytophilum*, antinuclear antibody test and rapid in‐clinic test for *E. canis/ewingi*, *A. phagocytophilum/platys*, *D. immitis*, and *B. burgdorferi* (SNAP4Dx Idexx, Hoofddorp, The Netherlands) were performed in all cases. Microscopic agglutination test (MAT) for *Leptospira* spp. and PCR for *Babesia* spp. were carried out if exposure was suspected, as previously reported.^39^, ^40^, ^41^ All tests, except for the MAT (carried out at the National Reference Centre for Animal Leptospirosis, Istituto Zooprofilattico Sperimentale della Lombardia e dell'Emilia‐Romagna, IZSLER, Bologna, Italy), were performed at the clinical pathology laboratory of the Department of Veterinary Medical Sciences of the University of Bologna.

### 2.5 Statistical analysis

An a priori power analysis was performed to estimate the minimum number of dogs to be included in the study groups. Based on our preliminary results (unpublished data), the hypothesis was that at different time points, a higher percentage of dogs receiving the combined protocol (90%) would achieve a satisfactory (partial or complete) hematological recovery, compared with dogs receiving only steroids (50%). Considering a 1:2 case ratio between single and combined‐treatment groups, and in order to achieve 80% statistical power and 5% type I error rate (α), a minimum of 42 dogs had to be enrolled. Distribution of data were assessed by graphic evaluation and using the D'Agostino‐Pearson test. Data were reported using standard descriptive statistics as mean ± SD, or median and range (minimum‐maximum value), if normally or non‐normally distributed, respectively. Means difference and 95% confidence intervals (95% CIs) of endpoints data between and among treatment groups were reported. Clinical and laboratory data were compared among groups using the Student *t* test or the Mann Whitney *U* test (2‐groups comparison), or the Kruskall‐Wallis test with a compensated post‐hoc analysis (3‐group comparison) for continuous variables. Fisher exact test or Chi‐square test were used for categorical variables. Per protocol (PP) study sample included dogs completing the study without major protocol deviations and performing all the required serial evaluations. Hematological recovery was assessed in the PP study sample and compared between dogs treated with glucocorticoid monotherapy (M‐group) and combined protocols (MC and MM‐group evaluated together), and among the 3 different therapeutic groups (M, MC, and MM‐group). Median dose of methylprednisolone during the period of treatment with this drug was calculated for each group. Length of hospitalization, frequency of complications, recurrence, and death were assessed in the overall study sample and compared. Overall survival time was calculated as the time interval between first day of hospitalization and IMHA‐related death. Death rate within therapeutic groups was measured at discharge, T60 and T365. Dogs deceased for IMHA‐unrelated causes were censored. Survival plots were generated according to Kaplan‐Meier product limit method and results compared with the Log‐rank test. An intention‐to‐treat analysis based on all grouped dogs randomized upon admission and regardless of their withdrawal from the treatment (*n* = 2), was also performed for the primary outcome (hematological recovery). Analyses were carried out using a commercially available statistical software (MedCalc Statistical Software version 20.011, MedCalc Software Ltd, Ostend, Belgium). The significance level was set at *P* < .05.

## 3 RESULTS

### 3.1 Demographics, clinical, and clinicopathological data

Fifty‐two dogs were diagnosed with IMHA during the study period. Four dogs were excluded because of aIMHA (*Ehrlichia* spp. infection *n* = 2; *L. infantum* infection *n* = 1; hemophagocytic histiocytic sarcoma *n* = 1); 5 dogs died before randomization. Forty‐three dogs were enrolled in the study. All these dogs tested negative for infectious diseases.

The study included 19/43 (44%) males (16 intact; 3 castrated), and 24/43 (56%) females (7 intact; 17 spayed). Median age was 7 years (range 0.5‐14 years) and median body weight was 10.5 kg (range 3.4‐52.2 kg). There were 26/43 (60%) purebred dogs and 17/43 (40%) cross‐breeds (Table S2). Median duration of clinical signs before admission was 2 days (range 0‐20 days). Most common clinical signs evaluated at T0 are presented in Table 1. Upon admission, anemia was moderate in 8/43 (19%) dogs, severe in 15/43 (35%), and very severe in 20/43 (46%); anemia was regenerative in 18/43 (42%) dogs; 28/43 (65%) dogs had leukocytosis and 18/43 (42%) had thrombocytopenia. Laboratory data collected upon admission are shown in Tables 2 and 3, and S3. Based on the Consensus Statement,^2^ 36/43 (84%) dogs achieved the diagnostic criteria for IMHA, including 3/43 (7%) dogs having only positive SAT after washing; the remaining 7/43 (16%) had only 1 of the proposed signs of immune‐mediated destruction associated with signs of hemolysis, and therefore had a supportive diagnosis of IMHA.

**TABLE 1:** Clinical findings recorded upon admission in dogs with naIMHA (*n* = 43) included in the study.

Clinical sign	Number of dogs (%)
Lethargy	43/43 (100%)
Pigmented urine	36/43 (84%)
Pale mucous membranes	35/43 (83%)
Anorexia	32/43 (74%)
Jaundice	19/43 (44%)
Diarrhea	8/43 (19%)
Fever (*T*° >39.2°C)	6/43 (14%)
Vomiting	5/43 (12%)
Polyuria and polydipsia	1/43 (2.5%)

**TABLE 2:** Apple_fast_ score, CHAOS and selected clinicopathological results in dogs with naIMHA (*n* = 43) included in the study and comparison of clinicopathological results among naIMHA dogs divided based on the therapeutic group and evaluated upon admission.

Variable	naIMHA dogs	M‐group	MC‐group	MM‐group	*P* value	Reference Interval
	*n* = 43	*n* = 16	*n* = 13	*n* = 14		
Apple_fast_ score	27 (16–39)	27 (19–39)	28 (16–32)	26.5 (17–33)	.59	Up to 50
Lactate (mmol/L)	2.3 (0.6–12.1)	2.5 (1.3–12.1)	2.15 (0.6–3.4)	2.3 (0.9–9.6)	.25	0–1 mmol/L
CHAOS	4 (0–7)	3.5 (1–7)	3 (0–7)	4 (1–7)	.22	Up to 7
Hematology						
HCT (%)	14.3 ± 5.7	14.3 ± 6.0	14.1 ± 6.0	14.4 ± 5.6	.92	37%–55%
MCV (fL)	73.3 (56.3–111.6)	80.2 (63.8–111.6)	73.9 (56.3–83.5)	69.8 (59.1–92.7)	.48	60–77 fL
MCHC (%)	34.4 (26.5–72.4)	33.9 (26.5–72.4)	34.5 (27.4–45.2)	35.45 (27.8–59.0)	.63	32.0%–38.0%
Reticulocytes (/mm^3^)	87 400 (500–564 500)	144 100 (500–395 700)	82 900 (1800–264 600)	102 250 (1600–564 500)	.46	≥120 000/mm^3^
WBC (/mm^3^)	19 440 (5910–71 160)	18 325 (8660–68 660)	18 130 (5910–56 120)	25 210 (8250–71 160)	.32	6000–17 000/mm^3^
Platelets (/mm^3^)	194 000 (6000–911 000)	212 500 (6000–911 000)	180 000 (66000–784 000)	224 500 (63000–834 000)	.65	160 000–500 000/mm^3^
Serum biochemistry						
ALT (U/L)	55 (10–7144)	56 (22–2212)	50 (33–700)	64 (10–7144)	.82	15–52 U/L
ALP (U/L)	320 (21–2782)	181 (21–954)	373 (66–1931)	424 (122–2782)	.24	12–180 U/L
GGT (U/L)	2.4 (0.1–11.8)	1.9 (0.1–7.9)	3.0 (0.1–11.8)	2.25 (0.1–9.6)	.67	0–5 U/L
Total bilirubin (mg/dL)	1.04 (0.16–19.31)	0.85 (0.21–18.07)	0.91 (0.16–12.93)	2.75 (0.25–19.31)	.25	0.07–0.33 mg/dL
Total protein (g/dL)	6.3 ± 0.6	6.2 ± 0.5	6.2 ± 0.9	6.2 ± 0.9	.30	5.6–7.3 g/dL
Albumin (g/dL)	2.71 ± 0.36	2.75 ± 0.23	2.91 ± 0.50	2.90 ± 0.50	.63	2.75–3.85 g/dL
Creatinine (mg/dL)	0.69 (0.45–2.11)	0.67 (0.51–1.60)	0.68 (0.49–1.22)	0.71 (0.45–2.11)	.68	0.75–1.40 mg/dL
Urea (mg/dL)	52 (12–267)	58 (18–123)	49 (25–74)	58 (12–267)	.33	17–48 mg/dL
Phosphate (mg/dL)	3.85 (2.20–8.56)	3.70 (2.88–8.56)	3.72 (2.20–5.60)	4.10 (2.90–7.14)	.59	2.65–5.40 mg/dL
Coagulation						
PT (s)	7.5 (5.5–24.0) *n* = 38	7.9 (5.7–24.0) *n* = 12	7.0 (6.0–13.7) *n* = 13	7.6 (5.5–11.2) *n* = 13	.61	5.0–7.5 s
aPTT (s)	12.2 (8.4–27.0) *n* = 38	12.3 (8.4–23.7) *n* = 12	11.0 (9.3–27.0) *n* = 13	12.2 (8.8–21.0) *n* = 13	.87	8.0–16.5 s
Fibrinogen (g/L)	3.75 (0.38–11.06) *n* = 38	2.78 (0.38–3.57) *n* = 12	2.74 (2.15–6.61) *n* = 13	2.60 (2.06–11.06) *n* = 13	.82	1.45–3.85 g/L

*Note*: Data are reported as median and range (min–max values) or mean ± SD, based on their distribution.

Abbreviations: ALP, alkaline phosphatase; ALT, alanine transaminase; aPTT, activated partial thromboplastin time; CHAOS, canine hemolytic anemic objective score; GGT, gamma‐glutamyl transferase; HCT, hematocrit; M therapeutic group, methylprednisolone therapeutic group; MC therapeutic group, methylprednisolone and cyclosporine therapeutic group; MCHC, mean corpuscular hemoglobin concentration; MCV, mean corpuscular volume; MM therapeutic group, methylprednisolone and mycophenolate mofetil therapeutic group; PT, prothrombin time; USG, urine specific gravity; WBC, white blood cells.

**TABLE 3:** Diagnostic criteria for naIMHA used in the study population.

Variable	Number of dogs (%)	M‐group	MC‐group	MM‐group	*P* value
Hematocrit value <37%	43/43 (100%)	16/16	13/13	14/14	1
Hyperbilirubinemia	38/43 (88%)	15/16	10/13	13/14	.30
Bilirubinuria ≥3 mg/dL (positive dipstick)	36/43 (84%)	13/16	11/13	12/14	.94
Spherocytosis	33/43 (77%)	12/16	8/13	13/14	.15
Positive SAT	33/43 (77%)	13/16	9/13	11/14	.73
Positive Coombs' test	24/36 (67%)	11/15	5/8	8/13	.77
Hemoglobinemia	25/43 (58%)	11/16	7/13	7/14	.54

*Note*: Data are expressed as frequency of presence of the selected variable.

Abbreviations: M‐group, methylprednisolone therapeutic group; MC‐group, methylprednisolone and cyclosporine therapeutic group; MM‐group, methylprednisolone and mycophenolate mofetil therapeutic group; naIMHA, non‐associative immune‐mediated hemolytic anemia; SAT, saline autoagglutination test.

### 3.2 Therapeutic groups and PP study sample

**FIGURE 1:**
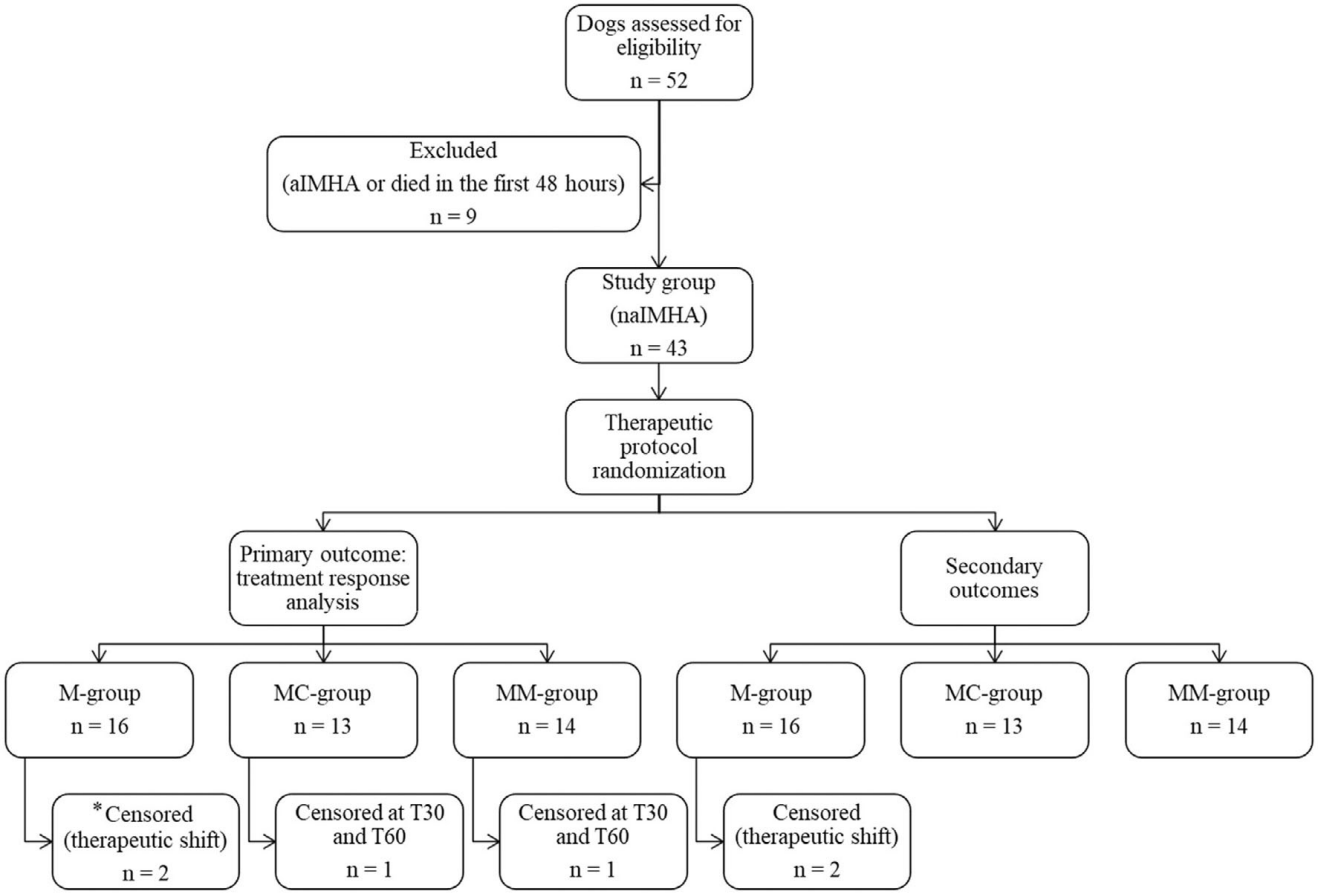
Flow chart of dogs with immune‐mediated hemolytic anemia (IMHA) included in the study: enrollment, randomization and comparison. aIMHA, associative immune‐mediated hemolytic anemia; naIMHA, non‐associative immune‐mediated hemolytic anemia; M‐group, methylprednisolone therapeutic group; MC‐group, methylprednisolone plus cyclosporine therapeutic group; MM‐group, methylprednisolone plus mycophenolate mofetil therapeutic group. Secondary outcomes: need for packed red blood cells transfusions, duration of hospitalization, number and severity of complications, frequency of relapse and mortality rate. *Assessed in the intention‐to‐treat analysis for primary outcome.

Sixteen out of 43 dogs were randomly assigned to M‐group, 13/43 to MC‐group and 14/43 to MM‐group. Therapeutic groups were not different for demographic data, clinicopathological variables, APPLE_fast_ score, CHAOS (Tables 2 and 3) and median dose of corticosteroids received throughout the study period (Table 4). Because of severe related adverse effects and complications, 5/41 dogs (12%; 3/13 in MC and 2/14 in MM group, respectively) had a faster tapering of glucocorticoids (median duration of therapy 80 days; range 49‐86). Two dogs in the M‐group, which had no improvement within the first 2 weeks of treatment were removed from the study; 41 dogs were included in the T14 PP study sample. Serial clinical and clinicopathological data were lost for 2 dogs after the first 2 weeks, and consequently 39 dogs were included in the T30 and T60 PP study sample. The flow chart of dogs' inclusion is shown in Figure 1.

**TABLE 4:** Comparison of clinical and clinicopathological endpoints, frequency of complications, and mortality among therapeutic groups.

Endpoint	M‐group	MC‐group	MM‐group	*P* value	M vs. MC	MC vs MM	M vs MM
					Mean difference (95% CI)	Mean difference (95% CI)	Mean difference (95% CI)
	*n* = 14	*n* = 13	*n* = 14				
Clinical and clinicopathological endpoints							
Total PRBC volume administered (mL/kg)	15 (0–84) *n* = 14	22 (0–112) *n* = 12	26.5 (5–79) *n* = 14	.14	7.7 (−14.3–29.7)	6.3 (−15.9–24.5)	14.1 (−4.0–32.2)
Corticosteroid treatment dose (median mg/kg/days of treatment)	1.25 (0.80–3.33) *n* = 14	1.05 (0.55–1.73) *n* = 12	1.14 (0.82–2.86) *n* = 13	.09	−0.5 (−1.0–0.0)	0.5 (0.0–0.1)	0.0 (−0.6–0.6)
Length of hospitalization (days)	8 (4–17) *n* = 14	8 (4–13) *n* = 12	9 (5–16) *n* = 14	.63	0.2 (−2.0–2.4)	0.8 (−1.4–3.0)	1.0 (−1.4–3.4)
Resolution of anemia (days)	45 (16–120) *n* = 10	37 (11–155) *n* = 12	76 (18–120) *n* = 7	.61	11.8 (−25.7–49.3)	8.1 (−36.6–52.8)	17.9 (−17.4–53.2)
Disappearance of spherocytosis (days)	30 (1–109) *n* = 11	47 (14–84) *n* = 8	29 (2–76) *n* = 9	.34	7.7 (−23.2–38.6)	−14.9 (−38.6–8.8)	−7.2 (−36.4–22.0)
Disappearance of positive SAT (days)	11 (1–79) *n* = 10	8 (1–52) *n* = 9	4 (2–14) *n* = 9	.65	−3.9 (−23.7–15.9)	−6.7 (−18.6–5.2)	−10.6 (−27.4–6.2)
Normalization of serum bilirubin concentration (days)	15 (2–60) *n* = 11	12 (3–74) *n* = 8	16 (4–47) *n* = 8	.74	−1.9 (−21.5–17.7)	−0.8 (−21.3–19.7)	−2.7 (−18.5–13.1)
Frequency of complications and relapse							
Anemia‐induced tissue hypoxia complications (number of dogs)	1/14	0/13	2/14	.36	7.1 (−16.4–31.4)	14.3 (−10.7–40.0)	7.2 (−19.2–33.5)
Thrombotic complications (number of dogs)	3/14	2/13	4/14	.70	6.0 (−24.2–34.4)	13.2 (−18.5–41.5)	7.2 (−23.9–36.7)
Infectious complications (number of dogs)	4/14	5/13	4/14	.81	9.9 (−23.4–40.9)	9.9 (−23.4–40.9)	0.0 (−31.0–31.0)
Iatrogenic hyperadrenocorticism complications (number of dogs)	3/14	6/13	4/14	.36	24.8 (−10–53.1)	17.6 (−17.1–47.5)	7.2 (−23.9–36.7)
Minor complications (number of dogs)	6/14	8/13	5/14	.38	18.7 (−17.1–48.6)	25.8 (−10.6–54.2)	7.1 (−26.2–38.4)
Major complications (number of dogs)	8/14	6/13	8/14	.80	11.0 (−23.8–42.4)	11.0 (−23.8–42.4)	0.0 (−32.6–32.6)
Relapse of IMHA (number of dogs)	2/14	0/13	1/14	.36	14.3 (−10.7–40.0)	7.1 (−16.4–31.4)	7.2 (−19.2–33.5)
Hematological recovery							
CHR at T14 (number of dogs)	0/14	0/13	0/14	.98	0.0 (−21.5–22.8)	0.0 (−22.8–21.5)	0.0 (−21.5–21.5)
PHR at T14 (number of dogs)	2/14	0/13	1/14	.36	14.3 (−10.7–40.0)	7.1 (−16.4–31.4)	7.2 (−19.2–33.5)
CHR at T30 (number of dogs)	1/13	2/13	0/13	.33	7.7 (−20.2–35.3)	15.4 (−9.9–42.2)	7.7 (−16.0–33.3)
PHR at T30 (number of dogs)	3/13	2/13	2/13	.84	7.7 (−23.0–37.0)	0.0 (−29.0–29.0)	7.7 (−23.0–37.0)
CHR at T60 (number of dogs)	3/13	5/13	3/13	.60	15.4 (−18.8–45.4)	15.4 (−18.8–45.4)	0.0 (−31.0–31.0)
PHR at T60 (number of dogs)	1/13	3/13	3/13	.49	15.4 (−14.2–43.3)	0.0 (−31.0–31.0)	15.4 (−14.2–43.3)
Mortality							
Death at discharge (number of dogs)	1/14	0/13	2/14	.36	7.1 (−16.4–31.4)	14.3 (−10.7–40.0)	7.2 (−19.2–33.5)
Death at T60 (number of dogs)	3/14	0/13	6/14	**.03** *	21.4 (−5.3–47.6)	42.8 (11.5–67.4)	21.4 (−12.4–49.6)
Death at T365 (number of dogs)	3/14	0/13	7/14	**.009** *	21.4 (−5.3–47.6)	50.0 (17.5‐73.2)	28.6 (−6.4–55.6)

*Note*: Data are reported as median, range (min − max values), mean difference (%) and 95% confidence interval (95% CI). Significant *P* values are reported in bold. Corticosteroid treatment dose refers to the median dose of methylprednisolone administered during the period of treatment with this drug.

Abbreviations: CHR, complete hematological recovery; IMHA, immune‐mediated hemolytic anemia; M therapeutic group, methylprednisolone therapeutic group; IMHA, immune‐mediated hemolytic anemia; MC therapeutic group, methylprednisolone and cyclosporine therapeutic group; MM therapeutic group, methylprednisolone and mycophenolate mofetil therapeutic group; PHR, partial hematological recovery; PRBC, packed red blood cell; SAT, saline agglutination test.

* Significantly different from MC and MM groups.

### 3.3 Transfusion and medication requirements, and hospitalization

Thirty‐eight dogs out of 41 (93%) received at least 1 transfusion; 36/41 (88%) dogs received PRBCs (median volume administered, 18 mL/kg/dog, range 0‐112 mL/kg). There was no difference between single‐line and combined therapy, nor among therapeutic groups regarding the number of transfusion treatments (*P* = .86 and *P* = .90, respectively), and the median ml/kg of PRBCs administered (*P* = .10 and *P* = .14, respectively). Median dose of MMF and cyclosporine, administered during the period of treatment with these drugs, were 14 mg/kg/day (range 13.1–17.2 mg/kg/day), and 5 mg/kg/day (range 4.4–6.4 mg/kg/day), respectively. Details of supportive medications are reported in Table S4. Median length of hospitalization was 8 days (range 4–17 days), with no difference documented between dogs receiving only steroids and dogs treated with combined therapy (*P* = .38), nor among the 3 therapeutic groups (*P* = .63). Dog characteristics, including median dose of methylprednisolone administered, and secondary outcomes are summarized in Tables 4 and 5.

**TABLE 5:** Comparison of clinical and clinicopathological endpoints, frequency of complications, and case fatality rate between methylprednisolone alone and combined therapy treatment groups.

Endpoint	M‐group	MC + MM‐group	M vs MC + MM	*P* value
			Mean difference (95% CI)	
Clinical and clinicopathological endpoints				
Total PRBC volume administered (mL/kg)	15 (0–84) *n* = 14	22 (0–112) *n* = 27	11.4 (−5.9–28.7)	.10
Corticosteroid treatment dose (median mg/kg/days of treatment)	1.25 (0.80–3.33) *n* = 14	1.07 (0.55–2.86) *n* = 27	−0.2 (−0.7–0.3)	.29
Length of hospitalization (days)	8 (4–17) *n* = 14	8 (4–16) *n* = 27	0.6 (−1.3–2.5)	.38
Resolution of anemia (days)	45 (16–120) *n* = 10	60 (11–155) *n* = 19	14.8 (−17.3–46.9)	.58
Disappearance of spherocytosis (days)	30 (1–109) *n* = 11	33 (2–84) *n* = 17	−0.2 (−23.3–22.9)	.57
Disappearance of positive SAT (days)	11 (1–79) *n* = 10	5 (1–52) *n* = 18	−7.2 (−20.9–6.5)	.42
Normalization of serum bilirubin concentration (days)	15 (2–60) *n* = 11	14 (3–74) *n* = 16	−2.3 (−16.9–12.3)	.56
Frequency of complications and relapse				
Anemia‐induced tissue hypoxia complications (number of dogs)	1/14	2/27	0.3 (−24.6–17.3)	.97
Thrombotic complications (number of dogs)	3/14	6/27	0.8 (−27.8–23.9)	.95
Infectious complications (number of dogs)	4/14	9/27	4.7 (−25.2–29.9)	.75
Iatrogenic hyperadrenocorticism complications (number of dogs)	3/14	10/27	15.6 (−14.8–38.9)	.31
Minor complications (number of dogs)	6/14	13/27	5.2 (−24.9–33.2)	.75
Major complications (number of dogs)	8/14	14/27	5.2 (−24.9–33.2)	.75
Relapse of IMHA (number of dogs)	2/14	1/27	10.6 (−7.2–36.4)	.22
Hematological recovery				
CHR at T14 (number of dogs)	0/14	0/14	0.0 (−21.5–21.5)	1
PHR at T14 (number of dogs)	2/14	1/27	10.6 (−7.2–36.4)	.22
CHR at T30 (number of dogs)	1/13	2/26	0.0 (−26.2–17.6)	1
PHR at T30 (number of dogs)	3/13	4/26	7.6 (−15.9–36.3)	.56
CHR at T60 (number of dogs)	3/13	8/26	7.7 (−23.0–32.0)	.61
PHR at T60 (number of patients)	1/13	6/26	15.3 (−13–35.3)	.24
Case fatality rate				
Death at discharge (number of dogs)	1/14	2/27	0.3 (−24.6–17.3)	.97
Death at T60 (number of dogs)	3/14	6/27	0.8 (−27.8–23.9)	.95
Death at T365 (number of dogs)	3/14	7/27	4.5 (−24.6–27.8)	.75

*Note*: Corticosteroid treatment dose refers to the median dose of methylprednisolone administered during the period of treatment with this drug. Data are reported as median, range (min − max values), mean difference (%), and 95% confidence interval (95% CI).

Abbreviations: CHR, complete hematological recovery; HCT, hematocrit; IMHA, immune‐mediated hemolytic anemia; M therapeutic group, methylprednisolone therapeutic group; IMHA, immune‐mediated hemolytic anemia; MC therapeutic group, methylprednisolone and cyclosporine therapeutic group; MM therapeutic group, methylprednisolone and mycophenolate mofetil therapeutic group; NR, non‐responders; PHR, partial hematological recovery; PRBC, packed red blood cell.

### 3.4 Treatment response

**FIGURE 2:**
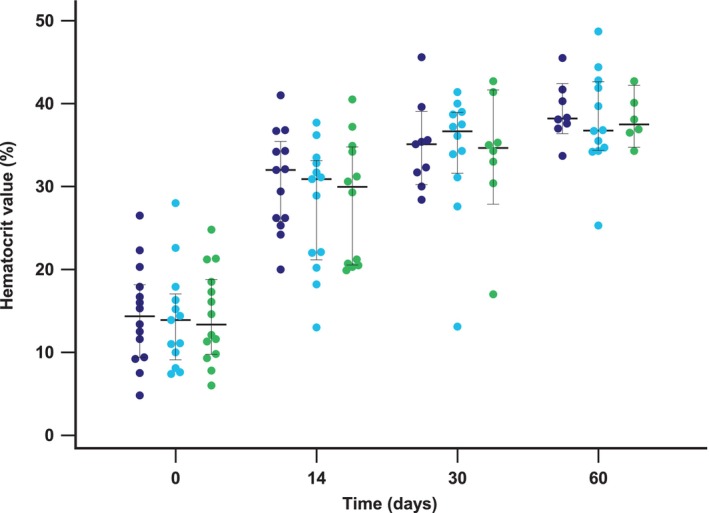
Clustered multiple variable graph reporting hematocrit value at different time points for 3 groups of treatment. Blue dots represent methylprednisolone therapeutic group; light blue dots represent methylprednisolone plus cyclosporine therapeutic group; green dots represent methylprednisolone plus mycophenolate mofetil therapeutic group. Central line and whiskers represent the median and the 95% Confidence Interval for median, respectively.

In PP study sample at T14, no dogs had a CHR and only 3/41 (7%) had a PHR; at T30, 10/39 (26%) dogs achieved the hematological endpoint (8% CHR, 18% PHR). Finally, at T60, 18/39 (46%) dogs reached a CHR or PHR (28% and 18%, respectively). Frequency of responders was not significantly different between dogs in M‐group and dogs treated with combined protocols (MC and MM‐groups), nor among the 3 groups, at T14 (*P =* .22; *P* = .36, respectively), T30 (*P =* .60; *P* = .58, respectively) and T60 (*P =* .17; *P* = .28, respectively; Tables 4 and 5). The same hematological comparisons were also not significantly different in the intention‐to‐treat analysis (Data S5 and S6). Changes of HCT values over time among 3 therapeutic groups were reported in Figure 2.

### 3.5 Complications and relapse

Eight dogs out of 41 (20%) did not show any complications; 11/41 (26%) dogs had minor complications, 14/41 (34%) dogs experienced major complications, and 8/41 (20%) showed both major and minor complications. Thirteen out of 41 (32%) dogs developed infections (sepsis, *n* = 3; multidrug resistant urinary tract infection, *n* = 3; multiple skin abscess, *n* = 2; cholangitis, *n* = 1; endocarditis, *n* = 1; septic arthritis, *n* = 1; pyelonephritis, *n* = 1; pneumonia *n* = 1), 12/41 (29%) dogs experienced IMHA‐related complication (9/41 had thrombotic complication, 3/41 had hypoxic events), and 1/41 (2%) dog died suddenly without a clearly identifiable cause on day 14;4/41 (10%) dogs faced both infectious and thrombotic complications. Frequency of major complications was not significantly different between steroids alone and combined therapy groups (*P* = .75), nor among M, MC, and MM‐group (*P* = .80).

Two dogs in the M‐group and 1 dog in MM‐group (3/41 dogs, 7%) had a relapse of IMHA after 115, 243, and 131 days, respectively. Relapse rate was not significantly different between single and combined therapy and among different treatment groups (*P* = .22 and *P* = .36 respectively), (Tables 4 and 5).

### 3.6 Case fatality rate

**FIGURE 3:**
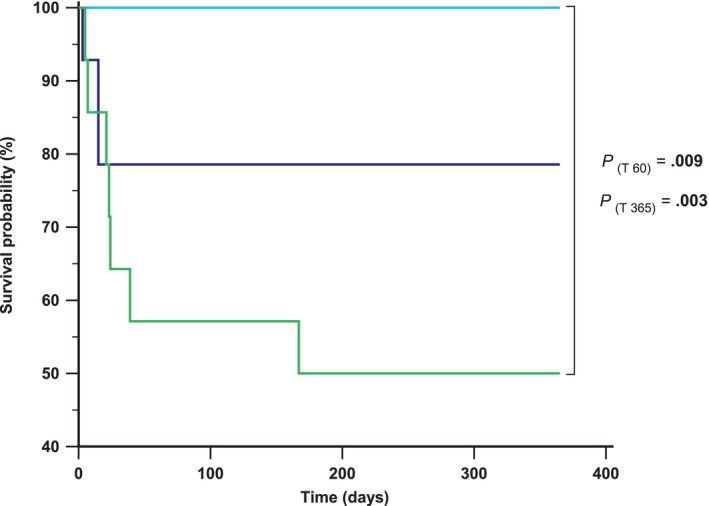
Kaplan‐Meier survival curves evaluating survival for dogs with naIMHA divided based on the 3 therapeutic groups. Blue line represents methylprednisolone therapeutic group (M‐group); light blue line represents methylprednisolone plus cyclosporine therapeutic group (MC‐group); green line represents methylprednisolone plus mycophenolate mofetil therapeutic group (MM‐group). A shorter survival was detected for MM‐group if compared with methylprednisolone and MC‐group at T60 (*P* = .009) and at T365 (*P* = .003).

The survival rate in this study was 93% (38/41 dogs) at discharge and 78% (32/41 dogs) at T60. At data analysis closure (T365), 31/41 (75%) dogs were alive and 10/41 (25%) had died because of IMHA‐related causes (*n* = 4; anemia‐induced tissue hypoxia, *n* = 3; thrombotic event, *n* = 1), treatment‐related complications (infection, *n* = 5) and unknown cause (*n* = 1). Two dogs (20%) were humanely euthanized because of IMHA complications (severe pneumonia, *n* = 1; thrombotic disease, *n* = 1). Moreover, 5/10 dogs (50%) died within the first 2 weeks (3 died during hospitalization, 2 died soon after discharge), whereas the remaining 5/10 (50%) dogs died after 21, 23, 24, 39, and 167 days from admission, respectively. When comparing the survival rate between single‐line and combined therapy groups no significant difference was detected at discharge (*P* = .97), at T60 (*P* = .95) and at T365 (*P* = .75). When cases were classified based on the 3 treatment groups, there was no difference in the case fatality rate at discharge (*P* = .36), while there was a significant difference at T60 (*P* = .03) and at T365 (*P* = .009). No differences emerged between M‐group and MM‐group (*P* = .22; *P* = .3), and M‐group and MC‐group (*P* = .08; *P* = .09), at T60 and T365, respectively; however, case fatality rate was significantly higher for dogs belonging to MM‐group compared with MC‐groups (*P* = .009 and *P* = .003) at the same 2 time‐points. Median survival time for MC‐group (365 days; 95% CI: 365–365) was significantly longer if compared with MM‐group (167 days; 95% CI: 21–167; *P* = .004), while no differences were detected with the median survival time of the M‐group (264 days; 95% CI: 205–365; *P* = .08). Kaplan‐Meier survival curves are shown in Figure 3.

## 4 DISCUSSION

Our study evaluated the effectiveness of different immunosuppressive protocols for the treatment of naIMHA in dogs, and identified no difference in response in dogs treated with a combined immunosuppressive protocol versus single agent corticosteroids. Currently, there is no consensus regarding the optimal immunosuppressive regimen or the benefit of adding a second‐line drug to glucocorticoids, for the treatment of IMHA in dogs, despite it being a common hematological disorder.^4^ In our trial, dogs were randomly assigned to receive either glucocorticoids alone, or combined with cyclosporine or mycophenolate mofetil. In contrast to our initial hypothesis, administration of a combined protocol did not significantly improve hematological recovery of enrolled dogs in any of the 3 timepoints considered, when compared with glucocorticoid monotherapy. The hematological recovery of our study group was relatively slow: specifically, a low percentage of dogs (18% and 8%, respectively) achieved a partial or complete recovery during the first month of treatment, and less than half of the study group fulfilled the criteria for complete or PHR after 2 months of treatment. Treatment‐related complications, including gastrointestinal bleeding and systemic inflammation leading to defective erythropoiesis, might have delayed the hematological recovery.

After 2 months of therapy, 21/39 dogs (54%) failed to reach any of the afore‐mentioned endpoints for hematological recovery. In addition to what suggested above, the high percentage of NR dogs could potentially indicate a poor effectiveness of the therapeutic protocols used in this study (selected dosages and time‐standardized tapering of immune suppressive treatment). It should be mentioned, however, that there is no consensus on the criteria to define hematological recovery in dogs with IMHA and that the ones chosen for this study are restrictive in their definition. Two retrospective studies compared the hematological response of different immunosuppressive protocols in dogs with IMHA. In the first study, 92/149 dogs treated with azathioprine and prednisolone were classified as improved or completely recovered at a median of 25 (range 2‐83) days after the initiation of therapy, but the cited study had some biases as underlined by the authors themselves (eg, incomplete description of treatment regimens, weak inclusion criteria, variable response evaluation time).^8^ A more recent study compared the immunosuppressive effectiveness of glucocorticoid monotherapy to combined protocols by assessment of time to PCV stabilization.^11^ In this study, the median number of days until the PCV plateaued (±2%), was 4 and no differences were highlighted between treatment groups. In both studies, median hematological recovery was shorter than in our study; however, less restrictive hematological endpoints were used precluding any meaningful comparison with our findings. Although the criteria used in our study conceptually approximate those used in human medicine,^42^, ^43^ further veterinary studies should define standard criteria to monitor the hematological recovery in dogs with IMHA, aiming at simplifying comparison of studies and applying published evidence to therapeutic decision‐making.

In our study, short‐term case fatality rate, evaluated at discharge, did not differ among the 3 therapeutic groups and between dogs treated with glucocorticoid monotherapy and those treated with combined protocols. This is likely because during the initial phase of the disease the benefit is mainly related to glucocorticoids administration, given the longer expected time of onset of cyclosporine and MMF effect in dogs.^44^, ^45^ With regard to the medium (T60) and long‐term (T365) case fatality rate, the comparison between steroids and combined therapy groups did not lead to a significant difference. However, when dogs were divided according to specific treatment group, combination of MMF and methylprednisolone was associated with a higher case fatality rate than cyclosporine and methylprednisolone, the therapeutic group with the lowest case fatality rate. A previous retrospective study failed to document a significant difference in the percentage of case fatality rate at 1 month or 1 year after discharge in dogs treated with single‐agent prednisolone versus combined protocols including cyclosporine.^3^ However, the same study was limited by incomplete description of treatment regimens. In the cited study, some dogs belonging to the cyclosporine group also received single injections of cyclophosphamide, which was potentially detrimental to long term prognosis.^3^, ^4^

In our study, the case fatality rate at 1 year after diagnosis was 25%, which is lower than previously reported,^1^, ^3^, ^28^, ^46^, ^47^, ^48^ but in line with more recent publications.^20^, ^49^ This finding could suggest that, to date, better outcomes can be achieved in dogs treated with standardized therapeutic approaches and undergoing supportive treatment strategies and strict monitoring. Among the non‐survivors (*n* = 10), 5 dogs died during the first 2 weeks after diagnosis and the majority of the others (*n* = 3) within the first month. Six dogs died because of thrombosis or infections, whereas 3 died because of the persistence of hemolysis and severe hypoxia. One dog died suddenly without an identified cause and following an apparent hematological stabilization, and a necropsy was not performed.

Among dead dogs, 7/10 were in the MM‐group and, of these, 4 died because of infections. The higher infectious‐related deaths occurred in the MM group compared with the other therapeutic groups could suggest that more caution is needed when MMF is administered as a second‐line immunosuppressive drug. Overall adverse effects were observed in 80% of our dogs, similarly to another recent study.^35^ Minor complications, such as polyuria and polydipsia, polyphagia, dermatological signs, and muscle atrophy, were observed in 46% of dogs, in line with previously reported adverse effects observed among dogs undergoing chronic glucocorticoid therapy.^50^, ^51^, ^52^, ^53^ Notably, almost half of the dogs exhibited major complications, which were mainly of infectious (32%), or thromboembolic (22%) origin. The frequency of complications highlighted in our study was similar in the different therapeutic groups. However, the relatively small number of cases could have biased these results and our findings should be further confirmed.

Currently there is no uniformity in predicting or objectifying the severity of naIMHA.^20^, ^54^ The APPLE_fast_ score has been used in hospitalized dogs with systemic inflammation demonstrating a highly accurate prediction of death in critically ill dogs,^55^, ^56^, ^57^ and it was applied in our study to categorize illness severity. This scoring system includes perfusion indices (such as blood lactate) and platelet count, which have already been mentioned as possible prognostic factors in dogs with IMHA.^28^ A more extended version of this score, the acute patient physiologic and laboratory evaluation, has been applied in dogs with IMHA recently.^58^ Illness‐severity in our study was also assessed using the CHAOS, previously associated with a higher risk of death during hospitalization in dogs with primary IMHA.^20^ The application of both scoring systems could improve the current limitations in clinical stratification of dogs with IMHA, allowing for a more objective comparisons during clinical trials.

There are limitations that should be considered in our study. Inclusion criteria were established before the recent American College of Veterinary Internal Medicine (ACVIM) Consensus Statement on the diagnosis of IMHA^2^ and were based on the documentation of anemia and at least 1 sign of immune‐mediated erythrocyte destruction in dogs with a suggestive clinical presentation. Seven dogs had only 1 of the proposed diagnostic criteria to assess the presence of RBCs autoantibodies. Based on these guidelines,^2^ the diagnosis of IMHA would only be supportive in these dogs. However, all of them had signs of hemolysis, an underlying cause of hemolytic anemia was not identified through rigorous search, and all responded to immunosuppression with a favorable course. Although the therapeutic protocols used in our study are in line with the recent guidelines, some aspects differ. First, methylprednisolone was used as first line drug because of the availability of an injectable solution, which is simpler to administer in the early stages of the disease when dogs do not tolerate oral administration. This molecule, maintained in all dogs after discharge through oral formulation, is commonly marketed in Europe and is described in the treatment of human autoimmune hemolytic anemia.^59^ Furthermore, methylprednisolone has a similar anti‐inflammatory potency to prednisolone and prednisone.^60^, ^61^, ^62^ The choice to use a lower dose of cyclosporine (2.5 mg/kg q12h), than that recommended in the ACVIM consensus statement,^4^ represent the second difference in the protocols set in our study. This dose is however still considered therapeutic according to previous IMHA studies.^3^, ^63^ These aspects, along with the lack of therapeutic drug monitoring could have reduced the potential to identify a beneficial effect of the combined protocols. Additionally, the lack of blinding might have resulted in biased estimates of treatment effects. Two cases in the M‐group were excluded from the analysis of the secondary outcomes and this approach might have influenced the results regarding this treatment group. No attempt was made to categorize IMHA cases into intravascular or extravascular, autoantibody class, acute or chronic, presence or absence of bone marrow compensation; as previously reported,^64^ these pathogenetic entities could have different therapeutic responses and outcome and might need ad hoc therapeutic and prognostic evaluations. Supportive therapy was not prospectively standardized although every treatment was supervised by the same medical team with standardized protocols used at our Institution. Finally, sample size was estimated hypothesizing a difference in hematological recovery between steroids alone and combined therapy groups of 40%, based on preliminary data. Based on our results, this difference was not reached a posteriori. For this reason, negative results arising from our study should be interpreted with caution.

In conclusion, dogs with naIMHA undergoing combined immunosuppressive therapy seem not to have a better hematological response compared with dogs treated with corticosteroids alone.

## CONFLICT OF INTEREST DECLARATION

Authors declare no conflict of interest.

## OFF‐LABEL ANTIMICROBIAL DECLARATION

Authors declare no off‐label use of antimicrobials.

## INSTITUTIONAL ANIMAL CARE AND USE COMMITTEE (IACUC) OR OTHER APPROVAL DECLARATION

Approved by the Scientific Ethical Committee for Animal Testing of the University of Bologna, ID744.

## HUMAN ETHICS APPROVAL DECLARATION

Authors declare human ethics approval was not needed for this study.

## Supporting information


**Data S1.** Supporting information.

**Data S2.** Supporting information.

**Data S3.** Supporting information.

**Data S4.** Supporting information.

**Data S5.** Supporting information.

**Data S6.** Supporting information.

## References

[1] Piek CJ . Canine idiopathic immune‐mediated haemolytic anaemia: a review with recommendations for future research. Vet Q. 2011;31(3):129‐141. 22029883 10.1080/01652176.2011.604979

[2] Garden OA , Kidd L , Mexas AM , et al. ACVIM consensus statement on the diagnosis of immune‐mediated hemolytic anemia in dogs and cats. J Vet Intern Med. 2019;33(2):313‐334. 30806491 10.1111/jvim.15441 PMC6430921

[3] Swann JV , Skelly BJ . Evaluation of immunosuppressive regimens for immune‐mediated haemolytic anaemia: a retrospective study of 42 dogs. J Small Anim Pract. 2011;52(7):353‐358. 21668886 10.1111/j.1748-5827.2011.01074.x

[4] Swann JV , Garden OA , Fellman CL , et al. ACVIM consensus statement on the treatment of immune‐mediated hemolytic anemia in dogs. J Vet Intern Med. 2019;33(3):1141‐1172. 30847984 10.1111/jvim.15463 PMC6524099

[5] Weinkle TK , Center SA , Randolph JF , Warner KL , Barr SC , Erb HN . Evaluation of prognostic factors, survival rates, and treatment protocols for immune‐mediated hemolytic anemia in dogs: 151 cases (1993‐2002). J Am Vet Med Assoc. 2005;226(11):1869‐1880. 15934255 10.2460/javma.2005.226.1869

[6] Piek CJ , Junius G , Dekker A , Schrauwen E , Slappendel RJ , Teske E . Idiopathic immune‐mediated hemolytic anemia: treatment outcome and prognostic factors in 149 dogs. J Vet Intern Med. 2008;22(2):366‐373. 18346140 10.1111/j.1939-1676.2008.0060.x

[7] Whitley NT , Day MJ . Immunomodulatory drugs and their application to the management of canine immune‐mediated disease. J Small Anim Pract. 2011;52(2):70‐85. 21265846 10.1111/j.1748-5827.2011.01024.x

[8] Piek CJ , Van Spil WE , Junius G , et al. Lack of evidence of a beneficial effect of azathioprine in dogs treated with prednisolone for immune‐mediated hemolytic anemia: a retrospective cohort study. BMC Vet Res. 2011;7(1):15. 21489250 10.1186/1746-6148-7-15 PMC3096914

[9] Swann JW , Skelly BJ . Systematic review of evidence relating to the treatment of immune‐mediated hemolytic anemia in dogs. J Vet Intern Med. 2013;27(1):1‐9. 23279007 10.1111/jvim.12028

[10] Wang A , Smith JR , Creevy KE . Treatment of canine immune‐mediated haemolytic anaemia with mycophenolate mofetil and glucocorticoids: 30 cases (2007 to 2011). J Small Anim Pract. 2013;54(8):399‐404. 23879827 10.1111/jsap.12107

[11] Weng J , Levy NA , Abbott HY , et al. Retrospective analysis of immunosuppressive and anti‐thrombotic protocols in nonassociative immune mediated hemolytic anemia in dogs. J Vet Intern Med. 2023;37(2):528‐536. 36809664 10.1111/jvim.16652 PMC10061171

[12] Piek C . Immune‐mediated hemolytic anemias and other regenerative anemias. In: Ettinger JE , Feldman EC , eds. Textbook of Veterinary Internal Medicine. 8th ed. St Louis, MO: Elsevier Saunders; 2015:2078‐2099.

[13] Day MJ , Mackin AJ . Immune‐mediated haematological disease. In: Day MJ , ed. Clinical Immunology of the Dog and the Cat. 2nd ed. London, UK: Manson Publishing Ltd; 2011:94‐120.

[14] Caviezel LL , Raj K , Giger U . Comparison of 4 direct Coombs' test methods with polyclonal antiglobulins in anemic and nonanemic dogs for in‐clinic or laboratory use. J Vet Intern Med. 2014;28(2):583‐591. 24433319 10.1111/jvim.12292 PMC4004353

[15] Villiers E . Disorders of erythrocytes. In: Villiers E , Ristić J , eds. BSAVA Manual of Canine and Feline Clinical Pathology. 3rd ed. Waterwalls business park, Quedgeley, GL: British Small Animal Veterinary Association; 2014:38‐66.

[16] Paes G , Paepe D , Meyer E , et al. The use of the rapid osmotic fragility test as an additional test to diagnose canine immune‐mediated haemolytic anaemia. Acta Vet Scand. 2013;55(1):74. 24160183 10.1186/1751-0147-55-74 PMC3816578

[17] Czock D , Keller F , Rasche FM , Hussler U . Pharmacokinetics and pharmacodynamics of systemically administered glucocorticoids. Clin Pharmacokinet. 2005;44(1):61‐98. 15634032 10.2165/00003088-200544010-00003

[18] Swann JW , Skelly BJ . Canine autoimmune hemolytic anemia: management challenges. Vet Med (Auckl). 2016;7:101‐112. 30050843 10.2147/VMRR.S81869 PMC6055891

[19] Hayes G , Mathews K , Doig G , et al. The acute patient physiologic and laboratory evaluation (APPLE) score: a severity of illness stratification system for hospitalized dogs. J Vet Intern Med. 2010;24(5):1034‐1047. 20629945 10.1111/j.1939-1676.2010.0552.x

[20] Goggs R , Dennis SG , Di Bella A , et al. Predicting outcome in dogs with primary immune‐mediated hemolytic anemia: results of a multicenter case registry. J Vet Intern Med. 2015;29(6):1603‐1610. 26473338 10.1111/jvim.13642 PMC4864895

[21] Random.org [Internet] . https://www.random.org.

[22] Fitzgerald E , Barfield D , Lee KC , et al. Clinical findings and results of diagnostic imaging in 82 dogs with gastrointestinal ulceration. J Small Anim Pract. 2017;58(4):211‐218. 28276120 10.1111/jsap.12631

[23] Weston PJ , Maddox TW , Hõim SE , Griffin S , Mesquita L . Diagnostic utility of abdominal ultrasound for detecting non‐perforated gastroduodenal ulcers in dogs. Vet Rec. 2022;190(1):e199. 33899941 10.1002/vetr.199

[24] Plumb DC . Omeprazole. In: Plumb DC , ed. Plumb's Veterinary Drug Handbook. 7th ed. Stockholm, WI: PharmaVet Inc.; 2011:2629‐2635.

[25] Goggs R , Bacek L , Bianco D , Koenigshof A , Li RHL . Consensus on the rational use of Antithrombotics in veterinary critical care (CURATIVE): domain 2‐defining rational therapeutic usage. J Vet Emerg Crit Care (San Antonio). 2019;29(1):49‐59. 30654415 10.1111/vec.12791

[26] Blais MC , Bianco D , Goggs R , et al. Consensus on the rational use of Antithrombotics in veterinary critical care (CURATIVE): domain 3‐defining antithrombotic protocols. J Vet Emerg Crit Care (San Antonio). 2019;29(1):60‐74. 30654416 10.1111/vec.12795

[27] Haskins SC . Hypoxemia. In: Silverstein DC , Hopper K , eds. Small animal critical care medicine. 2nd ed. St Louis, MO: Elsevier Saunders; 2015:81‐86.

[28] Ralph AG , Brainard BM . Hypercoagulable states. In: Silverstein DC , Hopper K , eds. Small animal critical care medicine. 2nd ed. St Louis, MO: Elsevier Saunders; 2015:541‐554.

[29] DeLaforcade A , Bacek L , Blais MC , et al. Consensus on the rationale use of Antithrombotics in veterinary critical care (CURATIVE): domain 1 – defining population at risk. J Vet Emerg Crit Care (San Antonio). 2019;29:37‐48. 30654424 10.1111/vec.12797

[30] Hauptman JG , Walshaw R , Olivier NB . Evaluation of the sensitivity and specificity of diagnostic criteria for sepsis in dogs. Vet Surg. 1997;26(5):393‐397. 9381665 10.1111/j.1532-950x.1997.tb01699.x

[31] Troìa R , Ciuffoli E , Vasylyeva K , et al. Circulating methemoglobin fraction in dogs with sepsis. Front Vet Sci. 2020;7:341. 32656253 10.3389/fvets.2020.00341 PMC7326004

[32] Huang HP , Yang HL , Liang SL , Lien YH , Chen KY . Iatrogenic hyperadrenocorticism in 28 dogs. J Am Anim Hosp Assoc. 1999;35(3):200‐207. 10333257 10.5326/15473317-35-3-200

[33] Whittemore JC , Mooney AP , Price JM , Thomason J . Clinical, clinicopathologic, and gastrointestinal changes from administration of clopidogrel, prednisone, or combination in healthy dogs: a double‐blind randomized trial. J Vet Intern Med. 2019;33(6):2618‐2627. 31593364 10.1111/jvim.15630 PMC6872608

[34] Stockham SL , Scott MA . Urinary system. In: Stockham SL , Scott MA , eds. Foundamentals of Veterinary Clinical Pathology. 2nd ed. Ames, Iowa: Blackwell Publishing; 2008:708‐871.

[35] Weingart C , Thielemann D , Kohn B . Primary immune‐mediated haemolytic anaemia: a retrospective long‐term study in 61 dogs. Aust Vet J. 2019;97(12):483‐489. 31454853 10.1111/avj.12875

[36] Tvedten H . Laboratory and clinical diagnosis of anemia. In: Weiss DJ , Wardrop J , eds. Schalm's Veterinary Hematology. 6th ed. Iowa, MO: Wiley‐Blackwell; 2010:152‐161.

[37] Couto CG . Anemia. In: Nelson RW , Couto CG , eds. Small Animal Internal Medicine. 5th ed. St Louis, MO: Elsevier Saunders; 2014:1201‐1219.

[38] Ni J , Zhu W , Whang Y , et al. A reference chart for clinical biochemical tests of hemolyzed serum samples. J Clin Lab Anal. 2021;35(1):23561. 10.1002/jcla.23561 PMC7843283 32881061

[39] Schuller S , Francey T , Hartmann K , et al. European consensus statement on leptospirosis in dogs and cats. J Small Anim Pract. 2015;56(3):159‐179. 25754092 10.1111/jsap.12328

[40] Troìa R , Balboni A , Zamagni S , et al. Prospective evaluation of rapid point‐of‐care tests for the diagnosis of acute leptospirosis in dogs. Vet J. 2018;237:37‐42. 30089543 10.1016/j.tvjl.2018.05.010

[41] Farwell GE , LeGrand EK , Cobb CC . Clinical observations of babesia gibsoni and babesia canis infections in dogs. J Am Vet Med Assoc. 1982;180:507‐511. 7061333

[42] Hill QA , Hill A , Berentsen S . Defining autoimmune hemolytic anemia: a systematic review of the terminology used for diagnosis and treatment. Blood Adv. 2019;3:1897‐1906. 31235526 10.1182/bloodadvances.2019000036 PMC6595261

[43] Jäger U , Barcellini W , Broome CM , et al. Diagnosis and treatment of autoimmune hemolytic anemia in adults: recommendations from the first international consensus meeting. Blood Rev. 2020;41:100648. 31839434 10.1016/j.blre.2019.100648

[44] Boothe DM . Immunomodulators or biological response modifiers: introduction and miscellaneous agents. In: Boothe DM , ed. Small Animal Clinical Pharmacology and Therapeutics. 2nd ed. St Louis, MO: Elsevier Saunders; 2012:1150‐1192.

[45] Mulligan C , Seo CS , Kaplan B , et al. Oral mycophenolate mophetil, dose escalation trial assessing adverse effects and pharmacodynamic responses in normal dogs. Proceedings of the 34th ACVIM Congress. Maryland, USA: National Harbor; 2017.

[46] Reimer ME , Troy GC , Warnick LD . Immune‐mediated hemolytic anemia: 70 cases (1988‐1996). J Am Anim Hosp Assoc. 1999;35(3):384‐391. 10493413 10.5326/15473317-35-5-384

[47] Carr AP , Panciera DL , Kidd L . Prognostic factors for mortality and thromboembolism in canine immune‐mediated hemolytic anemia: a retrospective study of 72 dogs. J Vet Intern Med. 2002;16(5):504‐509. 12322697 10.1892/0891-6640(2002)016<0504:pffmat>2.3.co;2

[48] Goggs R , Boag AK , Chan DL . Concurrent immune‐mediated hemolytic anaemia and severe thrombocytopenia in 21 dogs. Vet Rec. 2008;163(11):323‐327. 18791206 10.1136/vr.163.11.323

[49] Bestwick JP , Sharman M , Whitley NT , et al. The use of high‐dose immunoglobulin M‐enriched human immunoglobulin in dogs with immune‐mediated hemolytic anemia. J Vet Intern Med. 2022;36(1):78‐85. 34779044 10.1111/jvim.16315 PMC8783326

[50] McDonough AK , Curtis JR , Saag KG . The epidemiology of glucocorticoid‐associated adverse events. Curr Opin Rheumatol. 2008;20(2):131‐137. 18349741 10.1097/BOR.0b013e3282f51031

[51] Dye TL , Diehl KJ , Wheeler SL , Westfall DS . Randomized, controlled trial of budesonide and prednisone for the treatment of idiopathic inflammatory bowel disease in dogs. J Vet Intern Med. 2013;27(6):1385‐1391. 24112400 10.1111/jvim.12195

[52] Rhoades AC , Vernau W , Kass PH , Herrera MA , Sykes JE . Comparison of the efficacy of prednisone and cyclosporine for treatment of dogs with primary immune‐mediated polyarthritis. J Am Vet Med Assoc. 2016;248(4):395‐404. 26829271 10.2460/javma.248.4.395

[53] Masters AK , Berger DJ , Ware WA , et al. Effects of short‐term anti‐inflammatory glucocorticoid treatment on clinicopathologic, echocardiographic, and hemodynamic variables in systemically healthy dogs. Am J Vet Res. 2018;79(4):411‐423. 29583045 10.2460/ajvr.79.4.411

[54] Swann JW , Skelly BJ . Systematic review of prognostic factors for mortality in dogs with immune‐mediated hemolytic anemia. J Vet Intern Med. 2015;29(1):7‐13. 25586014 10.1111/jvim.12514 PMC4858088

[55] Holowaychuk MK , Hanel RM , Wood RD , et al. Prospective multicenter evaluation of coagulation abnormalities in dogs following severe acute trauma. J Vet Emerg Crit Care (San Antonio). 2014;24(1):93‐104. 24410816 10.1111/vec.12141

[56] Giunti M , Troìa R , Bergamini Famigli P , et al. Prospective evaluation of the acute patient physiologic and laboratory evaluation score and an extended clinicopathological profile in dogs with systemic inflammatory response syndrome. J Vet Emerg Crit Care (San Antonio). 2015;25(2):226‐233. 25427754 10.1111/vec.12257

[57] Wainberg SH , Brisson BA , Reabel SN , et al. Evaluation of risk factors for mortality in dogs with lung lobe torsion: a retrospective study of 66 dogs (2000‐2015). Can Vet J. 2019;60(2):167‐173. 30705452 PMC6340258

[58] Cuq B , Blois SL , Bédard C , et al. Serum interleukin 17 concentrations in dogs with immune‐mediated hemolytic anemia. J Vet Intern Med. 2021;35(1):217‐225. 33219716 10.1111/jvim.15977 PMC7848375

[59] Barcellini W , Zaninoni A , Giannotta JA , Fattizzo B . New insights in autoimmune hemolytic anemia: from pathogenesis to therapy stage. J Clin Med. 2020;9(12):3859‐3878. 33261023 10.3390/jcm9123859 PMC7759854

[60] Boothe DM , Mealey KA . Glucocorticoids and mineralocorticoids. In: Boothe DM , ed. Small Animal Clinical Pharmacology and Therapeutics. 2nd ed. St. Louis, MO: Saunders; 2012:1119‐1149.

[61] Notari L , Kirton R , Mills DS . Psycho‐Behavioural changes in dogs treated with corticosteroids: a clinical behaviour perspective. Animals (Basel). 2022;12(5):592‐604. 35268161 10.3390/ani12050592 PMC8909229

[62] Aharon MA , Prittie JE , Buriko K . A review of associated controversies surrounding glucocorticoid use in veterinary emergency and critical care. J Vet Emerg Crit Care (San Antonio). 2017;27(3):267‐277. 28449321 10.1111/vec.12603

[63] Helmond SE , Polzin DJ , Armstrong PJ , Finke M , Smith SA . Treatment of immune‐mediated hemolytic anemia with individually adjusted heparin dosing in dogs. J Vet Intern Med. 2010;24(3):597‐605. 20384956 10.1111/j.1939-1676.2010.0505.x

[64] Goggs R , Rishniw M . Developing randomized clinical trials to evaluate treatment effects in canine IMHA. J Vet Emerg Crit Care (San Antonio). 2016;26(6):763‐765. 27723948 10.1111/vec.12546

