# Supplementary material for: Methylprednisolone alone or combined with cyclosporine or mycophenolate mofetil for the treatment of immune‐mediated hemolytic anemia in dogs, a prospective study
Source: J Vet Intern Med. 2024 Jul 3;38(5):2480–94. doi: 10.1111/jvim.17122 (PMC11423485; doi:10.1111/jvim.17122)
Supplement: Supplementary file 1 — Data S1. Supporting information. [file JVIM-38-2480-s004.docx]

Supplementary information 1. Diagnostic schedule for study dogs.

| **Time point** | **CBC** | **PCV** | **Blood lactate** | **SAT exam** | **Serum biochemistry** | **CRP** | **AT activity** | **Urine dipstick** | **UPC §** |
| --- | --- | --- | --- | --- | --- | --- | --- | --- | --- |
| T0  (day 0) | X | X | X | X | X | X | X | X |  |
| T2  (day 2) | X | X | X | X |  |  |  | X |  |
| T3  (day 3) | X | X | X | X | X |  | X | X |  |
| T4  (day 4) | X | X | X | X |  |  |  | X |  |
| T5  (day 5) | X | X | X | X |  |  |  | X |  |
| T6  (day 6) | X | X | X | X |  |  |  | X |  |
| T7  (day 7) | X | X | X | X | X | X* | X* | X |  |
| T14  (day 14) | X |  | X | X* | X | X* | X* | X | X* |
| T30  (day 30) | X |  | X | X* | X | X* | X* | X | X* |
| T60  (day 60) | X |  | X | X* | X | X* | X* | X* | X* |
| T120  (day 120) | X |  | X | X* | X | X* | X* | X* | X* |
| T180  (day 180) | X |  |  |  | X |  |  |  |  |
| T365  (day 365) | X |  |  |  | X |  |  |  |  |

*Clinicopathological variables were measured out until normalization within the reference range.

§Urine protein-to-urine creatinine ratio quantification was performed from the disappearance of pigmented urine onwards at different time points.

AT activity, antithrombin activity; CRP, C-reactive protein; SAT, saline autoagglutination test; UPC, urine protein-to-urine creatinine ratio.
