# Supplementary material for: Methylprednisolone alone or combined with cyclosporine or mycophenolate mofetil for the treatment of immune‐mediated hemolytic anemia in dogs, a prospective study
Source: J Vet Intern Med. 2024 Jul 3;38(5):2480–94. doi: 10.1111/jvim.17122 (PMC11423485; doi:10.1111/jvim.17122)
Supplement: Supplementary file 2 — Data S2. Supporting information. [file JVIM-38-2480-s002.docx]

Supplementary information 2. Distribution of breeds in the study group of dogs with naIMHA.

| **Breed** | **Number of dogs (%)** |
| --- | --- |
| Maltese | 5/43 (11.6%) |
| Jack Russell Terrier | 3/43 (7%) |
| American Pitbull Terrier | 2/43 (4.6%) |
| Border Collie | 2/43 (4.6%) |
| Cavalier King Charles Spaniel | 2/43 (4.6%) |
| Other breeds  Miniature Poodle (n = 1)  Medium Poodle (n = 1)  Chihuahua (n = 1)  Dogo Argentino (n = 1)  Epagneul Breton (n = 1)  Griffon Bleu de Gascogne (n = 1)  Maremma Sheepdog (n = 1)  Pinscher (n = 1)  Miniature Schnauzer (n = 1)  Irish Setter (n = 1)  Springer Spaniel (n = 1)  Shih-Tzu (n = 1) | 12/43 (28%) |
| Mixed-breed | 17/43 (39.6%) |
