# Supplementary material for: Methylprednisolone alone or combined with cyclosporine or mycophenolate mofetil for the treatment of immune‐mediated hemolytic anemia in dogs, a prospective study
Source: J Vet Intern Med. 2024 Jul 3;38(5):2480–94. doi: 10.1111/jvim.17122 (PMC11423485; doi:10.1111/jvim.17122)
Supplement: Supplementary file 3 — Data S3. Supporting information. [file JVIM-38-2480-s003.docx]

Supplementary information 3. Clinicopathological results in dogs with naIMHA (n=43) included in the study. Data are reported as median and range (min – max values) or mean ± standard deviation, based on their distribution. Frequency data are also reported.

| **Variable** | **Results** | **Reference Interval** |
| --- | --- | --- |
| *Hematology* (n=43) | | |
| HCT (%) | 14.3 ± 5.7 | 37 – 55% |
| Hb (gr%) | 5.08 ± 1.90 | 12 – 18 gr% |
| RBC (/mm3) | 1913255 ± 805634 | 5500000 – 8500000/mm3 |
| MCV (fL) | 73.3 (56.3 – 111.6) | 60 – 77 fL |
| MCHC (%) | 34.4 (26.5 – 72.4) | 32 – 38% |
| RDW (%) | 20.0 (12.5 – 39.5) | 13.0 – 15.7% |
| Reticulocytes (/mm3) | 87400 (500 – 564500) | ≥120000/mm3 |
| WBC (/mm3) | 19440 (5910 – 71160) | 6000 – 17000/mm3 |
| Neutrophils (/mm3) | 15320 (4090 – 62426) | 3000 – 12000/mm3 |
| Lymphocytes (/mm3) | 2130 (340 – 12830) | 1000 – 4800/mm3 |
| Monocytes (/mm3) | 1640 (270 – 7078) | 100 – 1400/mm3 |
| Platelets (/mm3) | 194000 (6000 – 911000) | 160000 – 500000/mm3 |
| MPV (fL) | 17.5 (10.8 – 35.7) | 6.6 – 10.9 fL |
| *Serum chemistry* (n=43) | | |
| ALT (U/l) | 55 (10 – 7144) | 15 – 52 U/L |
| AST (U/l) | 68 (16 – 2535) | 15 – 62 U/L |
| ALP (U(l) | 320 (21 – 2782) | 12 – 180 U/L |
| GGT (U/l) | 2.4 (0.1 – 11.8) | 0 – 5 U/L |
| Total bilirubin (mg/dL) | 1.04 (0.16 – 19.31) | 0.07 – 0.33 mg/dL |
| Total protein (g/dL) | 6.3 ± 0.6 | 5.6 – 7.3 g/dL |
| Albumin (g/dL) | 2.71 ± 0.36 | 2.75 – 3.85 g/dL |
| Albumin-to-globulin ratio | 0.78 ± 0.16 | 0.75 – 1.35 |
| Creatinine (mg/dL) | 0.69 (0.45 – 2.11) | 0.75 – 1.40 mg/dL |
| Urea (mg/dL) | 52 (12 – 267) | 17 – 48 mg/dL |
| Phosphate (mg/dL) | 3.85 (2.20 – 8.56) | 2.65 – 5.40 mg/dL |
| Sodium (mEq/L) | 146 (129 – 150) | 143 – 151 mEq/L |
| Potassium (mEq/L) | 3.7 ± 0.6 | 3.8 – 5.0 mEq/L |
| Chloride (mEq/L) | 113 ± 3 | 108 – 118 mEq/L |
| Total Calcium (mg/dL) | 9.1 ± 0.6 | 9.3 – 11.0 mEq/L |
| Total iron (µg/dL) | 235 ± 128 | 50 – 230 µg/dL |
| TIBC (µg/dL) | 357 ± 80 | 240 - 440 µg/dL |
| TIBC saturation (%) | 61 (11 – 116) | 25 – 63% |
| CRP (mg/dL) | 19.83 (1.06 – 48.80) | 0 – 0.85 mg/dL |
| *Coagulation* (n=43) | | |
| PT (sec) | 7.5 (5.5 – 24.0) | 5.0 – 7.5 sec |
| aPTT (sec) | 12.2 (8.4 – 27.0) | 8.0 – 16.5 sec |
| Fibrinogen (g/L) | 3.75 (0.38 – 11.06) | 1.45 – 3.85 g/L |
| D-dimer (µg/ml) | 0.08 (0.01 – 3.83) | 0 – 0.26 µg/mL |
| AT activity (%) | 93 ± 18 | 105 – 166% |
| *Urinalysis* | | |
| USG (n=37) | 1035 ± 14 | > 1030 |
| UPC* (n=32) | 0.6 (0.1 – 5.6) | 0 – 0.5 |

ALP, alkaline phosphatase; ALT, alanine transaminase; aPTT, activated partial thromboplastin time; AST, aspartate transaminase; AT activity, antithrombin activity; CRP, C-reactive protein; GGT, gamma-glutamyl transferase; Hb, total hemoglobin; HCT, hematocrit; MCHC, mean corpuscular hemoglobin concentration; MCV, mean corpuscular volume; MPV, mean platelet volume; naIMHA, non-associative immune-mediated hemolytic anemia; PT, prothrombin time; RBC, red blood cells; RDW, red blood cells distribution width; TIBC, total iron binding capacity; UPC, urine protein-to-urine creatinine ratio; USG, urine specific gravity; WBC, white blood cells.

* Quantification of the UPC value was carried out after resolution of macroscopic pigmenturia
