# Supplementary material for: Methylprednisolone alone or combined with cyclosporine or mycophenolate mofetil for the treatment of immune‐mediated hemolytic anemia in dogs, a prospective study
Source: J Vet Intern Med. 2024 Jul 3;38(5):2480–94. doi: 10.1111/jvim.17122 (PMC11423485; doi:10.1111/jvim.17122)
Supplement: Supplementary file 4 — Data S4. Supporting information. [file JVIM-38-2480-s006.docx]

Supplementary information 4. Supportive medications administered to patients during hospitalization, after discharge, or both.

| **Medication** | **Number of dogs (%)** | **M-group** | **MC-group** | **MM-group** |
| --- | --- | --- | --- | --- |
| Omeprazole | 8/43 (19%) | 3/16 | 3/13 | 2/14 |
| Doxycycline* | 13/43 (30%) | 3/16 | 5/13 | 5/14 |
| Unfractionated heparin | 41/43 (95%) | 15/16 | 12/13 | 14/14 |
| Clopidogrel | 41/43 (95%) | 15/16 | 12/13 | 14/14 |
| Fresh frozen plasma | 2/43 (5%) | 1/16 | 1/13 | 0/13 |

M-group, methylprednisolone therapeutic group; MC-group, methylprednisolone plus cyclosporine therapeutic group; MM-group, methylprednisolone plus mycophenolate mofetil therapeutic group.

* Only administered pending infectious testing and discontinued after negative results.
