# Supplementary material for: Methylprednisolone alone or combined with cyclosporine or mycophenolate mofetil for the treatment of immune‐mediated hemolytic anemia in dogs, a prospective study
Source: J Vet Intern Med. 2024 Jul 3;38(5):2480–94. doi: 10.1111/jvim.17122 (PMC11423485; doi:10.1111/jvim.17122)
Supplement: Supplementary file 5 — Data S5. Supporting information. [file JVIM-38-2480-s005.docx]

Supplementary information 5. Comparison of hematological recovery in intention to treat analysis among the three different therapeutic groups. Data reported the number of dogs that have reached the outcome (partial response or complete response).

| **Timepoint** | **M-group**  **n = 16** | **MC-group**  **n = 13** | **MM-group**  **n = 14** | ***P* value** |
| --- | --- | --- | --- | --- |
| T14  (number of dogs) | 2/16 | 0/13 | 1/14 | .42 |
| T30  (number of dogs) | 4/15 | 4/13 | 2/13 | .63 |
| T60  (number of dogs) | 4/15 | 8/13 | 6/13 | .17 |

M-group, methylprednisolone therapeutic group; MC-group, methylprednisolone and cyclosporine therapeutic group; MM-group, methylprednisolone and mycophenolate mofetil therapeutic group; T14, 14 days after inclusion in the study; T30, 30 days after inclusion in the study; T60, 60 days after inclusion in the study
